# Depressive symptoms among Mexican adolescent girls in relation to iron status, anaemia, body weight and pubertal status: results from a latent class analysis

**DOI:** 10.1017/S1368980022001203

**Published:** 2022-05-18

**Authors:** Arli Guadalupe Zarate-Ortiz, Hans Verhoef, Alida Melse-Boonstra, Bo-Jane Woods, Elida Estefania Lee-Bazaldúa, Edith JM Feskens, Angelica Quiroga-Garza, Ana Carla Cepeda-Lopez

**Affiliations:** 1 Division of Human Nutrition and Health, Wageningen University & Research, Wageningen 6700 AK, The Netherlands; 2 Medical Research Council (MCR) Unit, The Gambia at London School of Hygiene & Tropical Medicine, Serrekunda, Gambia; 3 Access to Nutrition Initiative, Utrecht, The Netherlands; 4 Department of Nutrition, Universidad de Monterrey, San Pedro Garza García, Mexico; 5 Department of Psychology, Universidad de Monterrey, San Pedro Garza García, Mexico

**Keywords:** Depression, Adolescence, Iron deficiency, Anaemia, Overweight, Obesity, Puberty

## Abstract

**Objective:**

The study examined the association between depressive symptoms and iron status, anaemia, body weight and pubertal status among Mexican adolescent girls.

**Design:**

In this cross-sectional study, depressive symptoms were assessed by the 6-item Kutcher Adolescent Depression Scale, and latent class analysis (LCA) was used to identify and characterise groups of girls based on depressive symptoms. Iron status and inflammation were assessed using ferritin and soluble transferrin receptor, C-reactive protein and alpha-1-acid glycoprotein, respectively. Multiple logistic and linear regressions were applied to model class membership as a function of iron status, anaemia, body weight and pubertal status.

**Participants:**

We collected data from 408 girls aged 12–20 years.

**Setting:**

Public schools in northern Mexico.

**Results:**

LCA yielded three classes of depressive symptoms: 44·4 % of the adolescents were ‘unlikely to be depressed’, 41·5 % were ‘likely to be depressed’ and 14·1 % were ‘highly likely to be depressed’. Our analyses demonstrated that iron-deficient girls had greater odds of being ‘likely depressed’ (OR 2·01, 95 % CI 1·01, 3·00) or ‘highly likely depressed’ (OR 2·80, 95 % CI 1·76, 3·84). Linear regression analyses revealed that lower Hb concentrations and higher body weight increased the probability of being ‘likely depressed’. There was no evidence that depressive symptoms were associated with age at menarche and years since menstruation.

**Conclusions:**

This study shows that iron-deficient adolescent girls are more likely to suffer from depressive symptoms and that lower concentrations of Hb and higher body weight increased the probability of experiencing depressive symptoms.

Previous observational studies have reported an association between iron deficiency and depressive symptoms in children and adolescents^(1)^. Adolescence is a period of intensive brain remodelling, and iron has a role in various neurological functions like myelin production, synaptogenesis and production of neurotransmitters, that is serotonin, norepinephrine and dopamine^(2,3)^. Disrupted myelination and lower concentration of these neurotransmitters are common in depressed persons^(4,5)^. Randomised controlled trials in women with postpartum depression have shown that iron supplementation improves depressive symptoms in iron-deficient and non-iron-deficient women^(6)^. In addition, low Hb concentration has been associated with an increased odds of adult depression by 43 % (95 % CI 1·23, 1·65)^(4)^. Anaemia and depression share symptoms such as fatigue, which may be the result of altered cerebral oxygen transportation. However, little attention has been paid to the role of iron deficiency and anaemia in depression among adolescents, especially girls who recently started their menses.

The low-degree chronic inflammation produced by adiposity might also represent a risk factor for depression. Longitudinal studies showed that BMI at the age of 14 years predicted BMI and inflammatory markers at the age of 17 years, where inflammation was also associated with depressive symptoms^(7)^. In addition, depressive symptoms were associated with increased IL-6 responses among adolescents with higher BMI but not among those with lower BMI^(8)^. The link between high BMI and depressive symptoms might not be explained exclusively through the biologically plausible pathways of adiposity and inflammation but also by psychosocial pathways of self-esteem, body dissatisfaction and social support^(9–11)^. Furthermore, we showed earlier that overweight and obesity increase the risk for iron deficiency^(12)^.

After pubertal onset, prevalence of depression in girls is approximately twice that of boys^(13–15)^. In a survey among Mexican high school students, depressive symptoms were reported by 34 % and 18 % of female and male students, respectively^(9)^. Pubertal development may be the mechanism that underlies the gender difference in rates of depression. Clinical and epidemiological evidence suggests that fluctuations in hormonal concentrations, particularly in oestrogens, may influence the regulation of the hypothalamic–pituitary–adrenal axis and this may alter the neurotransmitter systems^(10,16)^. Anomalous hypothalamic–pituitary–adrenal axis function has been associated with the onset of depression in adolescents, and this effect seems to be dependent on the pubertal stage^(11)^. In addition, the rise of androgens during puberty, most notably dihydrotestosterone and testosterone, is involved in the hippocampus development, and larger hippocampal volume is associated with the risk of depression^(17)^. Thus, puberty is a critical period of development in which examining factors associated with depression is essential.

This study aimed to examine the association between iron status and depressive symptoms in Mexican adolescent girls. In addition, we also explored whether Hb concentration, body weight and pubertal onset were associated with depressive symptoms.

## Methods

### Participants

We conducted a cross-sectional study in the cities of Santa Catarina and Monterrey, in Northern Mexico, from September 2018 to January 2019. Adolescent girls aged 12–20 years from public schools were recruited, and written informed consent was obtained from the adolescents and their parents. Initially, we planned to conduct an intervention study among 162 iron-deficient participants. Since the prevalence of iron deficiency in Mexican adolescent girls was 36 % in urban areas, we determined that we would need to screen 450 adolescent girls to find a large enough number of iron-deficient participants for our intervention study. Hence, this dictated the sample size of this study. Exclusion criteria were diagnosis of systemic disease that may affect iron status, history of major surgery in the last month and regular use of medication (except contraceptives). After applying these criteria, a total of 408 adolescent girls were recruited for complete assessment. Five girls had incomplete questionnaires and therefore were excluded from this analysis.

### Measurements

#### Depressive symptoms

In the remainder of this paper, ‘depressive symptoms’ refer to symptoms experienced by adolescents, such as depressed mood, loss of interest, reduced energy leading to increased fatigability and diminished activity. To assess depressive symptoms, we used the 6-item Kutcher Adolescent Depression Scale (6-KADS)^(18)^ (Appendix 1), a questionnaire that has been translated from English to Spanish and has been validated for use in Latino adolescents^(19)^. The questionnaire was applied individually and as privately as possible by trained staff members. Six questions briefly describe the characteristic symptomatology of depression, with a Likert scale of four points (0 = ‘almost never’ to 3 = ‘all the time’). The overall score consisted of the sum of the score of all the items. Therefore, the total score ranges between 0 and 18. Traditionally, individuals are classified as not showing depressive symptoms if the overall score is 0–6 and as showing depressive symptoms if the score is ≥ 6.

### Iron status

A trained staff member drew venous blood samples for subsequent assessment of iron indicators (serum concentrations of ferritin and soluble transferrin receptor) and inflammatory markers (serum concentrations of C-reactive protein and alpha-1-acid glycoprotein). Blood samples were drawn at school facilities between 08.00 and 12.00 hours approximately; not all the participants had fasted; however, fasting has no implications on ferritin concentrations. Serum samples were stored at –80°C. Iron and inflammation concentrations were measured using a sandwich ELISA by the VitMin Lab, Willstaett, Germany^(20)^. Depleted iron stores were defined as serum concentrations of ferritin < 15 µg/l^(21)^. The values for soluble transferrin receptor reported by the VitMin Lab are in the same range as the RAMCO assay. Therefore, iron-deficient erythropoiesis was defined as soluble transferrin receptor concentration > 8·3 mg/l^(20)^.

#### Anaemia

Anaemia was defined by capillary blood Hb concentrations < 120 g/l^(22)^, measured by a HemoCue 201+ portable photometer (HemoCue AB.).

#### Anthropometric measurements

Body weight was measured on a calibrated platform scale with a bar (SECA 700), to the nearest 100 g, and with participants wearing light clothes. Height was measured in centimetres with the subject barefoot using a stadiometer (SECA 213). Both body weight and height were measured in duplicate and using standardised techniques. A third measure was done if the two measures differ by more than 500 g or 1 cm, respectively. BMI-for-age (BAZ) was used to classify weight status according to the WHO growth standards^(23)^. BAZ was categorised as obese (> 2 sd), overweight (> 1 sd), normal (–2 sd to 1 sd) and thin (< –2 sd).

#### Age at menarche

Self-reported age at menarche (first menstruation) was used as a proxy indicator for the onset of puberty.

#### Years since menarche

Self-reported age at menarche was subtracted from the chronological age.

### 2Data analysis

The statistical programme IBM SPSS 27.0 was used to calculate the descriptive statistics of general characteristics of the study population. Latent GOLD 5.1 (https://www.statisticalinnovations.com/latent-gold-5-1/) was used to undertake the latent class analysis (LCA). LCA is a statistical technique that aims to identify distinctive subgroups of people who share common characteristics so that people within the same subgroup have a similar scoring pattern on the measured variables^(24)^. To model the association between the different classes of depressive symptoms and the exposure variables (iron status, anaemia, body weight and pubertal onset), three main steps were involved^(25)^: (1) estimating the model with the optimal number of latent classes; (2) classifying the adolescent girls into one of the classes based on the model selected in step 1; and (3) examining the relationship between the classes and the exposure variables.

Responses to 6-KADS items from 403 Mexican adolescent girls were available and complete for LCA. First, explanatory LCA with 1–5 classes was conducted with the six 6-KADS items introduced as ordinal indicators. We selected the model with the lowest value for the Bayesian information criterion, which indicates the balance between model fit and model simplicity. It is a basic assumption from LCA that external variables are not correlated within the identified classes, which is known as conditional independence^(24)^. After identifying the optimal class solution, we examined the conditional independence between latent class indicators and the exposure variables by inspecting the bivariate residuals after including each exposure variable one by one (see online supplementary material, Supplemental Table 1). Bivariate residual values higher than 3·0 indicate a residual association between variables^(25)^. We concluded that BMI-for-age was correlated with item 3 and item 6 of the 6-KADS questionnaire. We re-estimated the model, including BMI-for-age as an active covariate, thus correcting the encountered effect between BMI-for-age and items 3 and 6. This correction resulted in a reduction in the Bayesian information criterion value. Second, we used posteriori classification to assign each of the adolescent girls to one of the three latent classes from the corrected model, and this classification information was saved. Third, we used two types of exposure variables, nominal (for iron deficiency and iron-deficient erythropoiesis) and continuous (for Hb, BMI z-scores, years since menstruation and age at menarche), which we modelled using a multinomial logistic regression and linear regression, respectively. We conducted separate models for each exposure variable with class membership as the dependent variable. Maximum likelihood adjustment was used to correct for classification error bias.

## Results

### General characteristics of the study sample

Data of 403 Mexican adolescent girls (mean age 15·2 (sd 1·8) years) were analysed, of whom 94 (23·3 %) had a 6-KADS score equal or higher to 6, indicating the evidence of depression. In total, 21·8 % of the participants suffered from anaemia and 10·9 % from iron deficiency. Overweight or obesity was present in almost half of the adolescents (42·7 %). Detailed descriptive statistics are shown in Table 1.

**Table 1:** Summary statistics of the total sample and the three latent classes of depressive symptoms

Variable	Total sample		‘Unlikely depressed’		‘Likely depressed’		‘Highly likely depressed’	
	*n*	%	*n*	%	*n*	%	*n*	%
*n*	403		187		159		57	
Age (years)*								
Mean	15·2		15·0		15·2		15·8	
sd	1·8		1·7		1·9		1·8	
Age at menarche (years)*								
Mean	11·8		11·8		11·7		12·0	
sd	1·3		1·4		1·2		1·5	
Prevalence of depressive symptoms (6-KADS score > 6)	94	23·3 %	0	0 %	37	23·3 %	57	100 %
Prevalence of anaemia	88	21·8 %	33	17·6 %	38	23·9 %	17	28·8 %
Prevalence of iron deficiency	44	10·9 %	16	8·6 %	19	11·9 %	9	15·8 %
Prevalence of iron deficiency erythropoiesis	14	3·5 %	7	3·7 %	6	3·8 %	1	1·8 %
BMI-for-age								
Normal (–2 sd to 1 sd)	228	56·6 %	119	63·6 %	78	49·1 %	31	54·4 %
Overweight (> 1 sd)	101	25·1 %	42	22·5 %	47	29·6 %	12	21·1 %
Obese (> 2 sd)	71	17·6 %	25	13·4 %	32	20·1 %	14	24·6 %
CRP (mg/l)†								
Median	0·3		0·2		0·3		0·4	
Q_25_, Q_75_	0·0, 1·1		0·0, 0·8		0·0, 1·9		0·0, 2·0	
AGP (g/l)†	0·6	0·5, 0·8	0·6	0·5, 0·8	0·7	0·5, 0·8	0·6	0·5, 0·8

Depressive symptoms if 6-KADS score was ≥ 6; anaemia was defined as Hb levels < 120 g/l; iron deficiency was defined as concentrations of serum ferritin < 15 µg/l; iron-deficient erythropoiesis was defined as soluble transferrin receptor concentration > 8·3 mg/l. CRP, AGP: serum concentrations of C-reactive protein and *α*_1_-acid glycoprotein.

* Mean values (sd).

† Median values (Q_25_, Q_75_) or *n* (%).

### Heterogeneity of depressive symptoms

The LCA analysis indicated three distinctive classes for the degree of depressive symptoms. When BMI-for-age was entered in the model, the Bayesian information criterion value changed from 4332·5 to 4321·3, indicating the most optimal balance between model fit and model simplicity, and was therefore the preferred solution for classification (Table 2). The percentages of individuals with self-reported scores of almost never in each of the six items of the 6-KADS questionnaire are shown in Table 3. Class 1 was the largest subgroup, constituting 44·4 % of the adolescent girls, who were ‘unlikely depressed’. Class 2 was labelled as ‘likely depressed’ because the response pattern reflected moderate occurrence (sometimes) for four out of six of the 6-KADS items. Class 3 was composed of 14·1 % of the adolescents and was described by a higher occurrence of depressive symptoms, with more adolescent girls reporting suicidal or self-harm ideation than the other two subgroups. Thus, the last class was labelled as ‘highly likely depressed’.

**Table 2:** Comparison of models with different number of classes derived from latent class analysis

Mexican adolescent girls (*n* 403)	BIC	AIC	Npar	L^2^	df	*P*
1-class model	4670·5	4598·5	18	999·3	385	< 0·001
2-class model	4344·7	4244·7	25	631·5	378	< 0·001
3-class model	4332·5	4204·5	32	577·3	371	0·31
4-class model	4356·5	4200·5	39	559·3	364	0·17
5-class model	4378·4	4194·5	46	539·3	357	0·61
3-class model*	**4321·3**	**4181·6**	**35**	**4111·6**	**365**	**0·39**

Bayesian information criterion (BIC) and Akaike information criterion (AIC) are both indicators of parsimony, that is the balance between model fit and model simplicity; the lower their value, the better this balance. Number of parameters (Npar); the lowest Npar indicates the model with the most parsimony. Degrees of freedom (df). Likelihood ratio chi-squared goodness-of-fit statistic (L^2^) indicates the amount of the association among the variables that remained unexplained after estimating the model; the lower the value, the better the fit of the model to the data. *P*-value from the bootstrap provides a more precise estimate by relaxing the assumption that the L^2^ statistic follows a chi-square distribution; *P*-values < 0·05 indicate no improvement over the previous model.

* Model with BMI-for-age as continuous covariate with direct effect on 6-KADS questions 3 and 6. Bold values designate the selected model, with the best balance between model fit and model simplicity.

**Table 3:** Description of the selected 3-class latent class model

Over the last week, how have you been ‘usually’ regarding the following items:		Latent classes			
	Total sample	‘Unlikely depressed’	‘Likely depressed’	‘Highly likely depressed’	*P*
Prevalence	100 %	44·4 %	41·5 %	14·1 %	
1. Low mood, sadness, feeling blah or down, depressed, just can’t be bothered					< 0·0001
Almost never		0·615	0·101	0·000	
Sometimes	0·315	0·379	0·754	0·294	
Almost all the time	0·139	0·005	0·143	0·554	
All the time	0·022	0·000	0·003	0·151	
2. Feelings of worthlessness, hopelessness, letting people down, not being a good person					0·010
Almost never	0·544	0·818	0·427	0·017	
Sometimes	0·355	0·178	0·527	0·407	
Almost all the time	0·069	0·004	0·046	0·347	
All the time	0·032	0·000	0·002	0·229	
3. Feeling tired, feeling fatigued, low in energy, hard to get motivated, have to push to get things done, want to rest or lie down a lot					0·036
Almost never	0·223	0·322	0·185	0·022	
Sometimes	0·474	0·559	0·431	0·340	
Almost all the time	0·228	0·115	0·322	0·303	
All the time	0·074	0·004	0·062	0·335	
4. Feeling that life is not very much fun, not feeling good when usually would feel good, not getting as much pleasure from fun things as usual					
Almost never	0·697	0·960	0·603	0·132	0·006
Sometimes	0·241	0·039	0·359	0·540	
Almost all the time	0·055	0·001	0·038	0·275	
All the time	0·007	0·000	0·000	0·053	
5. Feeling worried, nervous, panicky, tense, keyed up, anxious					
Almost never	0·385	0·548	0·326	0·029	0·009
Sometimes	0·434	0·407	0·496	0·342	
Almost all the time	0·124	0·033	0·145	0·353	
All the time	0·057	0·011	0·033	0·276	
6. Thoughts, plans or actions about suicide or self-harm					
Almost never	0·888	0·988	0·882	0·587	0·10
Sometimes	0·077	0·012	0·105	0·205	
Almost all the time	0·020	0·000	0·013	0·102	
All the time	0·015	0·000	0·000	0·106	

Values indicate conditional probabilities. For example, given the membership of class 1, the probability of responding ‘Almost never’ to the first item is 0·615. Entries are given as response probabilities in each item of the 6-KADS questionnaire. Low *P*-values indicate that the questionnaire item was useful to differentiate between class memberships.

### Associations of class membership and iron status

We examined the association between the three depressive symptoms classes with iron deficiency and latent classes of depression, and iron-deficient erythropoiesis and latent classes of depression using multiple logistic regression models adjusted for other potential confounders, including years since menstruation, BMI z-scores and markers of inflammation (Table 4). Compared to their peers with normal ferritin concentrations, iron-deficient girls (serum ferritin < 15 µg/l) had higher odds of being ‘likely depressed’ and ‘highly likely depressed’ (OR 2·01; 95 % CI 1·01, 3·00 and OR 2·80; 95 % CI 1·76, 3·84, respectively). In contrast, we found no evidence of an association between depressive symptoms and iron-deficient erythropoiesis (soluble transferrin receptor concentration > 8·3 mg/l).

**Table 4:** Probability of latent class membership for exposure variables: results obtained by multinomial logistic regression analysis

Exposure variable	Latent classes					*P*
	‘Unlikely depressed’	‘Likely depressed’		‘Highly likely depressed’		
		OR	95 % CI	OR	95 % CI	
Iron deficiency						
Iron deficiency (serum ferritin concentration < 15 µg/l)						
Yes	1·0 (reference)	(reference)		(reference)		
No	1·0 (reference)	1·51	0·59, 2·43	2·34	1·38, 3·30*	0·23
Iron deficiency (serum ferritin concentration < 15 µg/l)†						
Yes	1·0 (reference)	(reference)		(reference)		
No	1·0 (reference)	2·01	1·01, 3·00	2·80	1·76, 3·84	0·11
Iron deficiency erythropoiesis						
Iron-deficient erythropoiesis (sTfR concentration > 8·3 mg/l)						
No	1·0 (reference)	(reference)		(reference)		
Yes	1·0 (reference)	1·28	–0·19, 2·75	0·80	–1·24, 2·83	0·91
Iron-deficient erythropoiesis (sTfR concentration > 8·3 mg/l)†						
No	1·0 (reference)	(reference)		(reference)		
Yes	1·0 (reference)	0·70	–0·57, 1·97	0·62	–1·54, 2·78	0·83

sTfR, soluble transferrin receptor.

Values indicate OR, obtained by multinominal logistic regression analyses with class 1 (‘unlikely depressed’) as the reference category. *P*-values are based on Wald statistics. sTfR, CRP, AGP: serum concentrations of soluble transferrin receptor, C-reactive protein and *α*1-acid glycoprotein.

* Interpretation: girls with iron deficiency have 134·0 % higher odds of being ‘highly likely depressed’ compared with girls without iron deficiency.

† Iron deficiency model adjusted for years since menstruation, BMI z-scores, CRP and AGP. Iron-deficient erythropoiesis model adjusted for the same variable than iron deficiency model and age at menarche.

### Associations of class membership with Hb, body weight and puberty

The associations between depressive symptoms and Hb concentration, body weight and puberty are reported in Table 5. Each unit increment in Hb concentration (1 g/l) increased the probability of membership in class ‘unlikely depressed’ by 16 percentage points and reduced the probability of membership in class ‘likely depressed’ by 17 percentage points (*P* = 0·04). Each unit increment in BMI-for-age (1 sd) increased the probability of being ‘likely depressed’ by 22 % but reduced the probability of being ‘unlikely depressed’ by 18 % and ‘highly likely depressed’ by 4 %. Each increment in chronological age by 1 year increased the probability of being ‘highly likely depressed’ by 20 %. We assessed the association between puberty and depressive symptoms with two different indicators, years since menstruation and age at menarche, but we found no evidence of an association between puberty and depressive symptoms.

**Table 5:** Probability of latent class membership for exposure variables: results obtained by linear regression analysis

	Latent classes						
	‘Unlikely depressed’		‘Likely depressed’		‘Highly likely depressed’		
Exposure variable	*β*	se	*β*	se	*β*	se	*P*
Hb							
Intercept	−1·65	0·99	2·50	0·95	−0·85	0·98	
Hb (g/l)	0·16	0·08*	−0·17	0·07	0·01	0·07	0·04
Body weight							
Intercept	0·55	0·10	0·14	0·13	−0·69	0·13	
BMI z-scores	−0·18	0·07	0·22	0·09	−0·04	0·10	0·004
Pubertal onset							
Age							
Intercept	2·63	0·77	1·37	0·96	−4·00	1·18	
Age (years)	−0·14	0·05	−0·06	0·06	0·20	0·07	0·005
Years since menstruation†							
Intercept	2·44	1·01	1·55	< 0·001	−3·99	< 0·001	
Years since menstruation (years)	0·52	0·49	−0·06	0·06	−0·46	0·50	0·38
Age at menarche							
Intercept	0·83	0·76	0·39	0·93	−1·22	1·96	
Age at menarche (years)	−0·04	0·06	−0·01	0·08	0·04	0·10	0·85

Values indicate *β* estimates with standard errors, obtained by multinominal linear regression analyses. *P*-values are based on Wald statistics.

* For example: probability of being member of the class ‘unlikely depressed’ = –1·65 + 0·16 (Hb, g/l). Interpretation: each increment in Hb, g/l by 1 unit increases the probability of membership in class ‘unlikely depressed’ by 16 percentage points.

† Model adjusted for age at menarche and age.

## Discussion

Three distinct subtypes of depression were derived from the LCA, in which the severity of the symptoms was the source of heterogeneity. Our results indicate that depression was associated with iron deficiency, low Hb concentration, higher body weight and age. There was no association between depression and puberty.

In the current study, iron-deficient girls had a higher chance to be ‘likely depressed’ (OR 2·01, 95 % CI 1·01, 3·00) or ‘highly likely depressed’ (OR 2·80, 95 % CI 1·76, 3·84) compared with non-iron-deficient girls. These findings are in accordance with other cross-sectional studies indicating that depression scores were higher in women with iron deficiency (OR 2·84 95 % CI 1·24, 6·51)^(26)^ and adolescents with iron deficiency anaemia (OR 2·34, 95 % CI 1·58, 3·46)^(27)^. However, cross-sectional studies give no evidence of causality, and unfortunately, there is limited availability of longitudinal and intervention studies of iron status and depression among adolescents. A retrospective cohort study in Taiwan showed that 20-year-old women with iron deficiency anaemia had a 49 % increased risk of depression compared with women without iron deficiency anaemia^(28)^. In addition, randomised controlled trials in women with postpartum depression demonstrated that iron supplementation improves the depressive symptoms in iron-deficient and non-iron-deficient women^(6)^. Previous studies have reported the effects of iron on different neurological activities, such as myelination and monoamine metabolism^(29)^. Therefore, it is plausible that iron deficiency contributes to depression.

Another important finding was that a higher body weight was associated with being in the ‘likely depressed’ group and inversely associated with being ‘unlikely depressed’. Similarly, results from the National Survey of Health and Nutrition in Mexico (ENSANUT-2012) showed that adult women with obesity had higher odds (OR 1·28 95 % CI 1·07, 1·53) of having depression in comparison with normal-weight women^(30)^. These findings are consistent with results from a meta-analysis that concluded that children and adolescents with obesity were more likely to be depressed (pooled OR 1·34; 95 % CI 1·1, 1·64), but no association was observed for their peers with overweight^(31)^. Some longitudinal studies have shown that depressive symptoms at baseline were associated with obesity after 1 year follow-up in white, black and Hispanic American adolescents^(32)^, in adults^(33)^ and in women but not in men^(34)^. In contrast, baseline obesity did not predict follow-up depression^(32)^. Whether the association between body weight and depression is bidirectional is not entirely elucidated as it is complex and is mediated by multiple biological pathways and psychosocial factors.

Contrary to expectations, this study did not find evidence of an association between depression and pubertal development. A possible explanation for the inconsistency with other studies is the use of different indicators for pubertal stage^(35–38)^. Puberty comprises two distinct but overlapping processes, adrenarche (early stage) and gonadarche (later stage)^(39)^. A dramatic rise of steroid hormones marks adrenarche and is associated with physical changes that include increased skin oil and acne, skeletal maturation and pubic hair growth. Gonadarche instead is a gradual process that typically starts with breast development for girls and finalises shortly after menarche^(39)^. Thus, pubertal timing and pubertal status play a different role in the development of depressive symptoms. Another possible explanation for this might be that the mean age at menarche was similar in the three subgroups of depression, indicating that most girls were in comparable pubertal stages. Future investigations should include participants at different pubertal stages and consider using various indicators of pubertal status and pubertal timing to elucidate the link between puberty and mental health.

The use of LCA as a statistical technique to identify subgroups of depression is a major strength of this study. The use of categorical diagnostic constructs can result in the loss of valuable information about the diagnosis, because those who score just below the diagnostic threshold are regarded as non-cases. LCA overcomes this pitfall and has additional benefits for understanding the biological pathways and treatment opportunities by discriminating subtypes of depression.

There were some limitations to this study and should be considered when interpreting our results. Primarily, because of the observational nature of our study, there may exist unmeasured or residual confounding in the associations of interest. Furthermore, while our analysis adjusted for common demographic variables, other psychosocial indicators, which are likely to encompass the biological pathways that influence mental health, were not measured. Despite these limitations, our results are a starting point for future studies to investigate the role of nutritional status on mental health.

In conclusion, iron-deficient adolescent girls were more likely to suffer from depressive symptoms, and low Hb concentration and higher body weight increased the probability of depression. These findings suggest clinical trials to determine if nutritional status plays a role in depression and if improving nutritional status may alleviate some symptoms of depression. In addition, greater focus on screening and detecting depression in adolescent girls, especially among those with poor nutritional status, is required.

## Supplementary material

For supplementary material accompanying this paper visit https://doi.org/10.1017/S1368980022001203. click here to view supplementary material
