# Depressive symptoms among Mexican adolescent girls in relation to iron status, anaemia, body weight and pubertal status: results from a latent class analysis – CORRIGENDUM

**DOI:** 10.1017/S1368980025000618

**Published:** 2026-04-01

**Authors:** Arli Guadalupe Zarate-Ortiz, Hans Verhoef, Alida Melse-Boonstra, Bo-Jane Woods, Elida Estefania Lee-Bazaldúa, Edith JM Feskens, Angelica Quiroga-Garza, Ana Carla Cepeda-Lopez

The authors apologise for typographical errors in the above article.

In multiple instances it is stated that both multinomial logistic regression and linear regression were used for modelling. However, only logistic regression was utilised since the outcome for all models is dichotomic (class membership yes/no). This error is present on pages 408 (abstract), 410 (Methods) and 413 (Table 5). These errors do not affect the results, as logistic regression was originally employed, but was mistakenly labelled as linear regression.

Furthermore, there are errors in Table 4 under the category “Iron deficiency (serum ferritin concentration < 15 μg/l)” and “Iron deficiency erythropoiesis (sTfR concentration > 8·3 mg/l)†”. In both instances, the first row should be labelled “no”, and the second row should be labelled “yes”. For the same table, all analyses have been re-run, and the corrected values for the iron deficiency erythropoiesis adjusted model have been updated.

A corrected version of Table 4 is below:

**Table 4.** Probability of latent class membership for exposure variables: results obtained by multinomial logistic regression analysis

	Latent Class					
	“Unlikely depressed”	“Likely depressed”		“Highly likely depressed”		
		OR	95%CI	OR	95%CI	P
Exposure variable						
Iron deficiency						
Iron deficiency (serum ferritin concentration < 15 μg/l)						
No	1·0 (reference)	(reference)		(reference)		
Yes	1·0 (reference)	1.51	0.59, 2.43	2.34	1.38, 3.30*	0.23
Iron deficiency (serum ferritin concentration < 15 μg/l)†						
No	1·0 (reference)	(reference)		(reference)		
Yes	1·0 (reference)	2.01	1.01, 3.00	2.80	1.76, 3.84	0.11
Iron deficiency erythropoiesis						
Iron-deficient erythropoiesis (sTfR concentration > 8·3mg/l)						
No	1·0 (reference)	(reference)		(reference)		
Yes	1·0 (reference)	1.28	−0.19, 2.75	0.80	−1.24, 2.83	0.91
Iron-deficient erythropoiesis (sTfR concentration > 8·3mg/l)†						
No	1·0 (reference)	(reference)		(reference)		0.83
Yes	1·0 (reference)	1.67	−0.02, 3.35	1.66	−0.62, 3.95	

sTfR, soluble transferrin receptor.

Values indicate OR, obtained by multinominal logistic regression analyses with class 1 (‘unlikely depressed’) as the reference category. P-values are based on Wald statistics.

sTfR, CRP, AGP: serum concentrations of soluble transferrin receptor, C-reactive protein and α1-acid glycoprotein.

*Interpretation: girls with iron deficiency have 134·0 % higher odds of being ‘highly likely depressed’ compared with girls without iron deficiency.

†Iron deficiency model adjusted for years since menstruation, BMI z-scores, CRP and AGP. Iron-deficient erythropoiesis model adjusted for the same variable than iron deficiency model and age at menarche.
