# Supplementary material for: Depressive symptoms among Mexican adolescent girls in relation to iron status, anaemia, body weight and pubertal status: results from a latent class analysis
Source: Public Health Nutr. 2022 May 18;26(2):408–15. doi: 10.1017/S1368980022001203 (PMC13076068; doi:10.1017/S1368980022001203)
Supplement: Supplementary file 1 [file S1368980022001203sup.zip › S1368980022001203sup001.docx]

**Supplemental Table 1**: Bivariate residuals for the covariates in relation to the 3-class model of depressive symptoms among Mexican adolescent girls.

|  | Indicators | | | | | |
| --- | --- | --- | --- | --- | --- | --- |
| Covariate | **Question 1** | **Question 2** | **Question 3** | **Question 4** | **Question 5** | **Question 6** |
| Age (y) | 0.696 | 0.345 | 1.526 | 0.121 | 0.010 | 1.097 |
| Hemoglobin (g/dL) | 0.125 | 0.408 | 0.427 | 0.104 | 0.000 | 0.027 |
| Ferritin (µg/L) | 0.749 | 0.287 | 0.043 | 0.003 | 0.026 | 0.287 |
| sTfR (mg/L) | 0.158 | 1.860 | 0.015 | 0.190 | 1.957 | 0.344 |
| Age at menarche (y) | 0.902 | 0.229 | 0.022 | 0.041 | 0.028 | 2.536 |
| BMI-for-age (SD) | 0.002 | 0.000 | **3.716** | 0.518 | 1.203 | **2.859** |
| CRP (mg/L) | 0.502 | 0.914 | 0.018 | 0.372 | 0.027 | 0.011 |
| AGP (g/L) | 0.888 | 0.340 | 0.133 | 0.030 | 0.825 | 0.828 |

BVR obtained by including the covariates one by one in step-one 3-class model. Using a cut-off point of 3.0 we can conclude that BMI-for-age has direct effect on items 3 and 6. This suggests that the probability of having a pattern of responses for these items is conditional on BMI. Therefore, we need to include BMI-for-age in the model as covariate, and the two encountered direct effects. sTfR, CRP, AGP: serum concentrations of soluble transferrin receptor, C-reactive protein and α1-acid glycoprotein
