# Supplementary material for: Depressive symptoms among Mexican adolescent girls in relation to iron status, anaemia, body weight and pubertal status: results from a latent class analysis
Source: Public Health Nutr. 2022 May 18;26(2):408–15. doi: 10.1017/S1368980022001203 (PMC13076068; doi:10.1017/S1368980022001203)
Supplement: Supplementary file 1 [file S1368980022001203sup.zip › S1368980022001203sup002.pdf]

6-ITEM Kutcher Adolescent Depression Scale: KADS

NAME :____________________ DATE :__________________

OVER THE LAST WEEK, HOW HAVE YOU BEEN "ON AVERAGE" OR "USUALLY" REGARDING THE FOLLOWING

1. Low mood, sadness, feeling blah or down, depressed, just can't be bothered.

☐ a) Hardly Ever ☐ b) Much of the time ☐ c) Most of the time ☐ d) All of the time

2. Feelings of worthlessness, hopelessness, letting people down, not being a good person.

☐ a) Hardly Ever ☐ b) Much of the time ☐ c) Most of the time ☐ d) All of the time

3. Feeling tired, feeling fatigued, low in energy, hard to get motivated, have to push to get things done, want to rest or lie down a lot

☐ a) Hardly Ever ☐ b) Much of the time ☐ c) Most of the time ☐ d) All of the time

4. Feeling that life is not very much fun, not feeling good when usually would feel good, not getting as much pleasure from fun things as usual.

☐ a) Hardly Ever ☐ b) Much of the time ☐ c) Most of the time ☐ d) All of the time

5. Feeling worried, nervous, panicky, tense, keyed up, anxious.

☐ a) Hardly Ever ☐ b) Much of the time ☐ c) Most of the time ☐ d) All of the time

6. Thoughts, plans or actions about suicide or self-harm.

☐ a) Hardly Ever ☐ b) Much of the time ☐ c) Most of the time ☐ d) All of the time

TOTAL SCORE: ___________________

© 2008 Stan Kutcher

6 - item KADS scoring:

In every item, score:

a) Hardly Ever	= 0
b) Much of the time	= 1
c) Most of the time	= 2
d) All of the time	= 3

then add all 6 item scores to form a single Total Score.

Interpretation of total scores:

Total scores at or above 6	Suggest 'possible depression' (and a need for more thorough assessment).
Total scores below 6	Indicate 'probably not depressed'.

Reference

- LeBlanc JC, Almudevar A, Brooks SJ, Kutcher S: Screening for Adolescent Depression: Comparison of the Kutcher Adolescent Depression Scale with the Beck Depression Inventory, Journal of Child and Adolescent Psychopharmacology, 2002 Summer; 12(2):113-26.

Self-report instruments commonly used to assess depression in adolescents have limited or unknown reliability and validity in this age group. We describe a new self-report scale, the Kutcher Adolescent Depression Scale (KADS), designed specifically to diagnose and assess the severity of adolescent depression. This report compares the diagnostic validity of the full 16-item instrument, brief versions of it, and the Beck Depression Inventory (BDI) against the criteria for major depressive episode (MDE) from the Mini International Neuropsychiatric Interview (MINI). Some 309 of 1,712 grade 7 to grade 12 students who completed the BDI had scores that exceeded 15. All were invited for further assessment, of whom 161 agreed to assessment by the KADS, the BDI again, and a MINI diagnostic interview for MDE. Receiver operating characteristic (ROC) curve analysis was used to determine which KADS items best identified subjects experiencing an MDE.

Further ROC curve analyses established that the overall diagnostic ability of a six-item subscale of the KADS was at least as good as that of the BDI and was better than that of the full-length KADS. Used with a cut-off score of 6, the six-item KADS achieved sensitivity and specificity rates of 92% and 71%, respectively—a combination not achieved by other self-report instruments. The six-item KADS may prove to be an efficient and effective means of ruling out MDE in adolescents.

© 2008 Stan Kutcher
